# VCA nanobodies target N-WASp to reduce invadopodium formation and functioning

**DOI:** 10.1371/journal.pone.0185076

**Published:** 2017-09-22

**Authors:** Tim Hebbrecht, Isabel Van Audenhove, Olivier Zwaenepoel, Adriaan Verhelle, Jan Gettemans

**Affiliations:** Department of Biochemistry, Faculty of Medicine and Health Sciences, Rommelaere Campus, Ghent University, Ghent, Belgium; University of Illinois at Chicago, UNITED STATES

## Abstract

Invasive cancer cells develop small actin-based protrusions called invadopodia, which perform a primordial role in metastasis and extracellular matrix remodelling. Neural Wiskott-Aldrich syndrome protein (N-WASp) is a scaffold protein which can directly bind to actin monomers and Arp2/3 and is a crucial player in the formation of an invadopodium precursor. Expression modulation has pointed to an important role for N-WASp in invadopodium formation but the role of its C-terminal VCA domain in this process remains unknown. In this study, we generated alpaca nanobodies against the N-WASp VCA domain and investigated if these nanobodies affect invadopodium formation. By using this approach, we were able to study functions of a selected functional/structural N-WASp protein domain in living cells, without requiring overexpression, dominant negative mutants or siRNAs which target the gene, and hence the entire protein. When expressed as intrabodies, the VCA nanobodies significantly reduced invadopodium formation in both MDA-MB-231 breast cancer and HNSCC61 head and neck squamous cancer cells. Furthermore, expression of distinct VCA Nbs (VCA Nb7 and VCA Nb14) in PC-3 prostate cancer cells resulted in reduced overall matrix degradation without affecting MMP9 secretion/activation or MT1-MMP localisation at invadopodial membranes. From these results, we conclude that we have generated nanobodies targeting N-WASp which reduce invadopodium formation and functioning, most likely via regulation of N-WASp—Arp2/3 complex interaction, indicating that this region of N-WASp plays an important role in these processes.

## Introduction

Metastasis is the primary cause of cancer associated deaths. In this process cancer cells leave a primary tumour to disseminate through the entire body [1]. In doing so, these cancer cells are able to create secondary tumours throughout the body, a lethal process for the cancer patient in almost all cases [1]. In order to leave the primary tumour, cancer cells create small actin-based protrusions to facilitate their spreading [2]. Invadopodia are such malignant specialized structures known to enable cancer cells to invade through natural barriers [2, 3]. Invadopodium formation is found in highly invasive cancer cell lines [4] and has been evinced *in vivo* [3]. A mature invadopodium displays considerable proteolytic activity. More specifically, invadopodium-related degradation of the extracellular matrix (ECM) has been linked to extravasation and metastasis *in vivo* [3]. Inside an invadopodium, approximately 129 different proteins can be found [5]. They contribute to the aggressive nature of invadopodia in cancer. Before an invadopodium can be formed, assembly of a precursor is required. This precursor will kick-start actin polymerisation towards the plasma membrane and invadopodia will arise [6–9].

Activation of the Arp2/3 –N-WASp pathway, found to be important in invadopodium formation, has been observed in a broad spectrum of cancers [10]. Arp2/3 (actin related protein) is a protein complex existing of seven subunits, of which Arp2 and Arp3 are the only two proteins of the complex that are actin-related [11]. Once Arp2/3 is activated, those two subproteins will mimic two actin monomers to boost the actin polymerisation [11, 12].

The neural variant of WASp (N-WASp) is a ubiquitously expressed member of the Wiskott-Aldrich syndrome protein (WASP) family [8, 13, 14]. This family is known as nucleation-promoting factors of the Arp2/3 complex [15] and fills the gap between reorganisation of the actin cytoskeleton and signalling molecules [16, 17]. Because N-WASp is a scaffold protein, it has several domains through which it interacts with interaction partners [8, 13, 14]. Using its C-terminal VCA domain, N-WASp can directly interact with actin monomers and the Arp2/3 complex. The VCA domain consists of a verprolin-homology (V) domain (a.k.a. WASp homology 2 (WH2) domain), a cofilin-like or central (C) domain and an acidic (A) domain [18]. While the V domain is a binding site for actin monomers, the Arp2/3 complex binds the latter two parts (C and A domains) [14, 18–21]. It has been shown *in vitro* that N-WASps VCA domain is needed during the formation of the invadopodium precursor [6, 17, 22, 23]. Moreover, by bringing actin monomers and Arp2/3 closer together, N-WASp stimulates and enhances the actin polymerisation [24].

We generated alpaca nanobodies against the VCA domain of N-WASp (VCA Nb) to investigate the role of this region in formation of cancer cell invadopodia. Nanobodies are single-domain antibody fragments or, more specifically, the variable part of the heavy chain of Camelid heavy chain antibodies [25]. A nanobody is the smallest, natural, antigen binding fragment that completely retains its original binding affinity and specificity. Contrary to RNAi, intracellularly expressed nanobodies or so-called intrabodies can interfere with protein functions without silencing the whole protein. Moreover, intracellular nanobodies render the need for overexpression, siRNA or dominant negative mutants superfluous, which makes it able to study functions of endogenous proteins. By using VCA Nbs, we study the influence of N-WASps VCA domain in invadopodium formation and extracellular matrix degradation. Using breast, prostate and head and neck cancer cell lines that inducibly express these VCA nanobodies, we show that this N-WASp region indeed contributes to invadopodium formation and extracellular matrix remodelling, suggesting that it is involved in invadopodium based spreading of cancer cells.

## Materials and methods

### Antibodies and reagents

Rabbit polyclonal anti-N-WASp (H100) was obtained from Sigma (St. Louis, MO, USA) and Santa Cruz Biotechnology (Santa Cruz, CA, USA). Mouse monoclonal anti-actin clone C4 (0869100) was obtained from MP Biomedicals (Santa Ana, CA, USA). Rabbit monoclonal anti-ARPC2 antibody (ab133315) and anti-MMP14 (ab51074) were obtained from Abcam (Cambridge, UK). Rabbit monoclonal anti-MMP9 was obtained from Epitomics (Burlingame, CA, USA). Alexa Fluor-labeled secondary goat anti-rabbit or anti-mouse IgG antibodies were obtained from Molecular Probes (Eugene, OR, USA). Mouse monoclonal anti-V5 antibody, Alexa Fluor labeled phalloidin and Mitotracker Orange were purchased from Invitrogen (Merelbeke, Belgium). The synthetic VCA—hN-WASp peptide biotin-Ahx-^420^KKVEQNSRPVSCSGRDALLDQIRQGIQLKSVADGQESTPPTPAPTSGIVGALMEVMQKRSKAIHSSDEDEDEDDEEDFEDDDEWED^505^-NH_2_, used for raising nanobodies (see further), was chemically synthesized by Caslo (Denmark).

### Generation of VCA Nbs

Nbs were obtained in collaboration with the Vlaams Instituut voor Biotechnologie (VIB) Nanobody Service Facility (NSF). All animal work was performed by the VIB NSF and has PHS approved animal welfare assurance from OLAW (F16-00131(A5593-01)). Briefly, two alpacas were immunized by a subcutaneous injection of human VCA-N-WASp peptide on days 0, 7, 14, 21, 28 and 35. On day 39, anticoagulated blood was collected for lymphocyte preparation. A nanobody library was constructed by extracting total RNA from peripheral blood lymphocytes and screened for the presence of antigen-specific nanobodies. The obtained Nbs were subcloned into the phagemid vector pMECS. In order to isolate N-WASp nanobodies, several rounds of phage panning were performed. These experiments suggested that the phage population was enriched for antigen-specific phages from round 3 panning onwards. In total, 380 colonies from 3rd and 4th rounds were randomly selected and analyzed by ELISA for the presence of antigen-specific nanobodies in their periplasmic extracts. The positives ones were sequenced. Based on those sequence data, the positive colonies represented 5 different groups of nanobodies in each of the two alpacas.

### cDNA cloning

Nbs were cloned into mitochondrial outer membrane (MOM) V5 pcDNA3.1 His_6_ and pEGFP-N1 vectors (Clontech, Mountain View, CA, USA), as described before [26–28]. The nanobodies were subcloned by means of a Cold Fusion Cloning Kit (System Biosciences, Mountain View, CA, USA). The first subcloning was performed into V5 pcDNA3.1 His_6_ vector by using the following primers: forward 5’-TCGATTCTACGCGTACCGGTGCCCAGGTGCAGCTGCAGGAG-3’ and reverse 5’-GGTGATGATGACCGGCTAGCTGGAGACGGTGACCTG-3’. Cloning of the Nbs in the pEGFP-N1 vector was done using the following primers: forward 5’-CGAGCTCAAGCTTCGGCCACCATGCAGGTGCAGCTGCAGGAG-3’ and reverse 5’-GGCGACCGGTGGATCCTTGCTGGAGACGGTGACCTG-3’.

To obtain inducible Nb expressing cells, a third cloning of the EGFP-tagged VCA Nbs into the pLVX-TP vector (Clontech) was done using following primers: Forward 5’-TGGAGAAGGATCCGCGGCCGCGCCACCATGGCCCAGGTGCAGCTGCAGGAGTCTGGG-3’ and reverse 5’-CTACCCGGTAGAATTCTTACTTGTACAGCTCGTCCATGCC-3’.

### Recombinant nanobody and N-WASp production

BL21 cells were transformed with VCA Nbs in pMECS vector or N-WASp in pTrcHis TOPO. They were grown in TB (with 100 μg/ml ampicillin) at 37°C. After induction with 1 mM IPTG, incubation was performed at 28°C (overnight for nanobody production or 4 h for N-WASp production).

Nanobodies were extracted from the periplasm, where they are located due to an N-terminal PelB signal, by means of an osmotic burst of the outer membrane using TES buffer (0.2 M Tris, 0.5 mM EDTA, 0.5 M sucrose, pH 8.0). Next an IMAC and/or gel filtration was performed for a higher purification, as described before [29].

To extract recombinant N-WASp, pelleted cells were dissolved in lysis buffer (1% Triton, 20 mM Tris-HCl, 150 mM NaCl, 1 mM PMSF and 1 mM protease inhibitor, pH 7.5) supplemented with 200 μg/ml lysozyme and incubated during 30 min at room temperature. After sonication and pelleting, IMAC purification was performed.

### Cell culture, transfection and transduction

MDA-MB-231 (ATCC HTB-26), PC-3 (ATCC CRL-1435) and HEK293T (ATCC CRL-11268) cells were maintained at 37°C in a humidified 10% CO_2_ incubator, HNSCC61 cells were grown at 5% CO_2_. All cells (except PC-3) were grown in DMEM. We obtained the HNSCC61 cells from Prof. Dr. Weaver [30] (Vanderbilt University) in August 2011, who obtained the cells from Prof. Dr. Yarbrough [31–33]. PC-3 cells were grown in RPMI. All media (from Gibco Life Technologies (Grand Island, NY, USA) were supplemented with 10% foetal bovine serum, 10 μg/mL streptomycin and 10 IU/mL penicillin. As an exception, HNSCC61 required 20% fetal bovine serum and an extra addition of 0.4 μg/mL hydrocortisone (Sigma-Aldrich, St. Louis, MO, USA).

JetPrime (Polyplus Transfection Inc., New York, NY, USA) was used to perform transient expression according to the manufacturer’s protocol. For virus production HEK293T cells were transfected using the calcium phosphate method as described before [28]. Inducible stable cell lines were obtained by transduction of the VCA Nbs in the pLVXTP vector, making use of the Lenti-X Tet-On advanced system (Clontech, Mountain View, CA, USA) according to the manufacturer’s protocol. Expression was induced by addition of 500 ng/mL doxycycline.

### Pull down assays, immunoprecipitations and binding assay

Cells were lysed with ice-cold lysis buffer (1% Triton, 20 mM Tris-HCl, 150 mM NaCl, 1 mM PMSF and 1 mM protease inhibitor, pH 7.5) [27]. Protein concentrations were determined using the Bradford assay (Bio-Rad Laboratories, Hercules, CA, USA).

In the pull down assays to analyse binding between N-WASp and the 10 VCA Nbs, recombinant HA-tagged nanobodies were immobilized onto HA-agarose beads (Sigma-Aldrich, St. Louis, MO, USA) (1.5 h at 4°C). Next, 650 μg crude cell lysate was added and followed by incubation (1.5 h at 4°C).

In the pull down to analyse binding between N-WASp and VCA Nbs using EGFP-tagged VCA Nb expressing cancer cells, we performed overnight incubation of cell lysate with anti-GFP antibody (at 4°C), followed by an incubation with Protein G Sepharose beads (GE Healthcare) (1,5 h at 4°C).

In the binding assay, recombinant HA-tagged VCA Nbs, in a concentration range, were incubated with the synthetic biotin-tagged VCA domain (1.5 h at 4°C). Next, 550 μg crude MDA-MB-231 cell lysate was added, as source for Arp2/3 and actin, and an incubation was followed (1.5 h at 4°C). Last, an immobilization on STREPTactin beads (IBA, Göttingen, Germany) was performed (1h at 4°C).

After washing steps, elution of the beads was obtained by boiling for 5 minutes in Laemmli SDS sample buffer (65 mM Tris-HCl, 20% glycerol, 5% SDS, 0.2% bromophenol blue, 5% β-mercaptoethanol in Milli-Q, pH 6.8). This was followed by SDS-PAGE and Western blot analysis. The H100 N-WASp antibody from Santa Cruz Biotechnology was used to detect N-WASp on blot.

### Isothermal titration calorimetry (ITC)

ITC was performed as described before [27–29]. Binding of VCA Nbs to VCA peptide was measured by isothermal titration calorimetry (ITC) using a MicroCal VP-ITC MicroCalorimeter and MICROCAL ORIGIN software (Malvern Instruments Ltd, Malvern, UK). The recombinant VCA Nbs were dialyzed against 20 mM Tris buffer and 150 mM NaCl at pH 7,5. Briefly, 8,6 μM biotinylated VCA peptide was titrated once with 108 μM VCA Nb7 and once with 88 μM VCA Nb14. 46,91 μM VCA Nb2 and 5,6 μM VCA Nb13 was titrated with 46,91 μM and 56 μM biotinylated VCA peptide, respectively. The obtained values are expressed with the standard deviation.

### Immunofluorescence and microscopy

Cells were seeded on a 100 μg/ml gelatin (Sigma-Aldrich, St. Louis, MO, USA) or 50 μg/ml rat tail type I collagen (BD Biosciences, Franklin Lakes, NJ, USA) coated coverslip. Cells were fixed using 3% paraformaldehyde, permeabilized using 0.1% Triton for 5 min and neutralized in 0.75% glycin for 20 min. Next, a blocking step was performed using 1% bovine serum albumin (Sigma-Aldrich), followed by an incubation with primary antibodies (1 h at 37°C) and Alexa Fluor-conjugated secondary antibodies (30 min at room temperature). DAPI (0.4 μg/ml; Sigma) and Alexa Fluor labeled phalloidin (Invitrogen) were used to stain nuclei and actin filaments, respectively. Cells were mounted using VectaShield (Vector Laboratories, Burlingame, CA, USA). For imaging, a Zeiss Axiovert 200 M Apotome epifluorescence microscope equipped with a cooled CCD Axiocam camera (Zeiss x63 1.4-NA Oil Plan-Apochromat objective; Carl Zeiss, Oberkochen, Germany) and Axiovision 4.5 software (Zeiss) was used at room temperature.

### Matrix degradation

In matrix degradation assays, coverslips were coated with Cy3-labeled gelatin according to the manufacturer’s protocol (QCM Gelatin Invadopodia Kit, Millipore, Billerica, MA, USA). 24h after induction with doxycycline, cells were seeded and allowed to degrade the matrix for another 24h. Degradation is found on the places where black holes were present in the matrix underneath the cells. Two parameters were used to analyze the degradation. The parameter ‘degraded area per cell area’ was obtained by dividing the total degraded area by the total cell area per picture. The second parameter ‘degradation area per cell’ is determined by the total degradation area divided by the amount of cells per picture.

### Gelatin zymography

24 h after induction with 500 ng/ml doxycycline, cells were seeded in a 1 mg/ml collagen matrix which was allowed to polymerize for 1 h at 37°C. After approximately 20 h incubation in serum-free medium, equal amounts of conditioned medium proteins were analyzed on 10% SDS-PAGE without (for secretion levels) or with 0.1% gelatin (for activity). In the SDS-PAGE containing 0.1% gelatin, proteins were enabled to renature by removing SDS with 2% Triton X-100 washing buffer. To analyse the activity, digestion was allowed during an overnight incubation in MMP buffer (50 mM Tris and 10 mM CaCl_2_, pH 7.5). Band intensities were analyzed with ImageJ.

### MT1-MMP localization

Nanobody expression was induced 24 h before cells were seeded on a 100 μg/ml gelatin matrix. They were fixed, stained and imaged as described above. MT1-MMP containing invadopodia were count when MT1-MMP dots were overlapping F-actin dots. To analyse MT1-MMP, the amount MT1-MMP containing invadopodia was divided by the total amount of invadopodia for each cell.

### Image and statistical analysis

Image analysis (protein intensities, intensity profiles, quantifications) was performed with ImageJ (National Institutes of Health, Bethesda, MD, USA). Statistical analysis was performed with GraphPad Prism (GraphPad Software Inc., San Diego, CA, USA) using Kruskal-Wallis test and Dunns post tests with p = 0.05. Bar plots were made using mean and SEM.

## Results

### N-WASp recognising of VCA nanobodies

**Fig 1:**
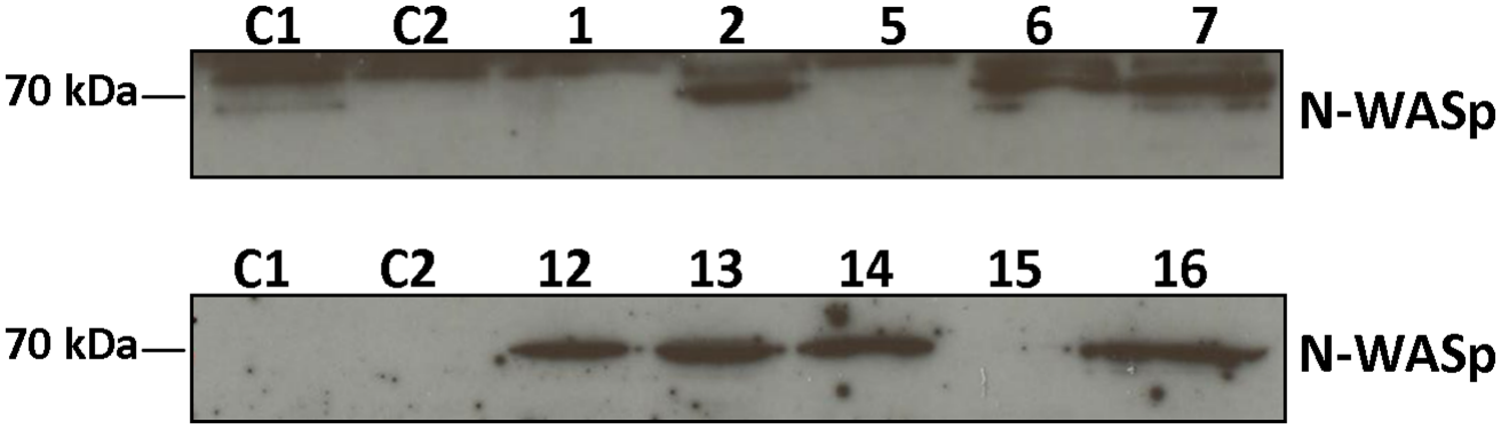
VCA nanobody—N-WASp binding. Pull down assay of endogenous N-WASp from MDA-MB-231 breast cancer cell lysate with HA-tagged VCA Nb (1, 2, 5, 6, 7 and 12, 13, 14, 15, 16) by means of anti-HA agarose beads. Two negative controls were included, anti-HA agarose beads were incubated with no nanobody (C1) or with a cortactin NTA nanobody against the N-terminal acidic (NTA) region of cortactin (C2). N-WASp was blotted and detected by anti-N-WASp antibody. To see the pull down of the 4 selected VCA Nbs using HNSCC61 cancer cells, which express EGFP-tagged VCA Nbs, see S1 Fig.

Initially, 16 different nanobodies were identified through phage display following immunization with chemically synthesized N-WASp VCA domain (human N-WASp K420-D505), lymphocyte collection and library construction. Based on their amino acid sequence, 10 different groups could be discerned. One nanobody was chosen from each group for biochemical characterisation. To detect antigen-nanobody binding *in vitro*, recombinant HA-tagged nanobodies were used in a pull down assay. MDA-MB-231 breast cancer cell lysate was used as a source of endogenous N-WASp. Seven nanobodies out of 10 bound with the endogenous N-WASp (Fig 1).

### Mitochondrial outer membrane anchoring and intracellular displacement of N-WASp

**Fig 2:**
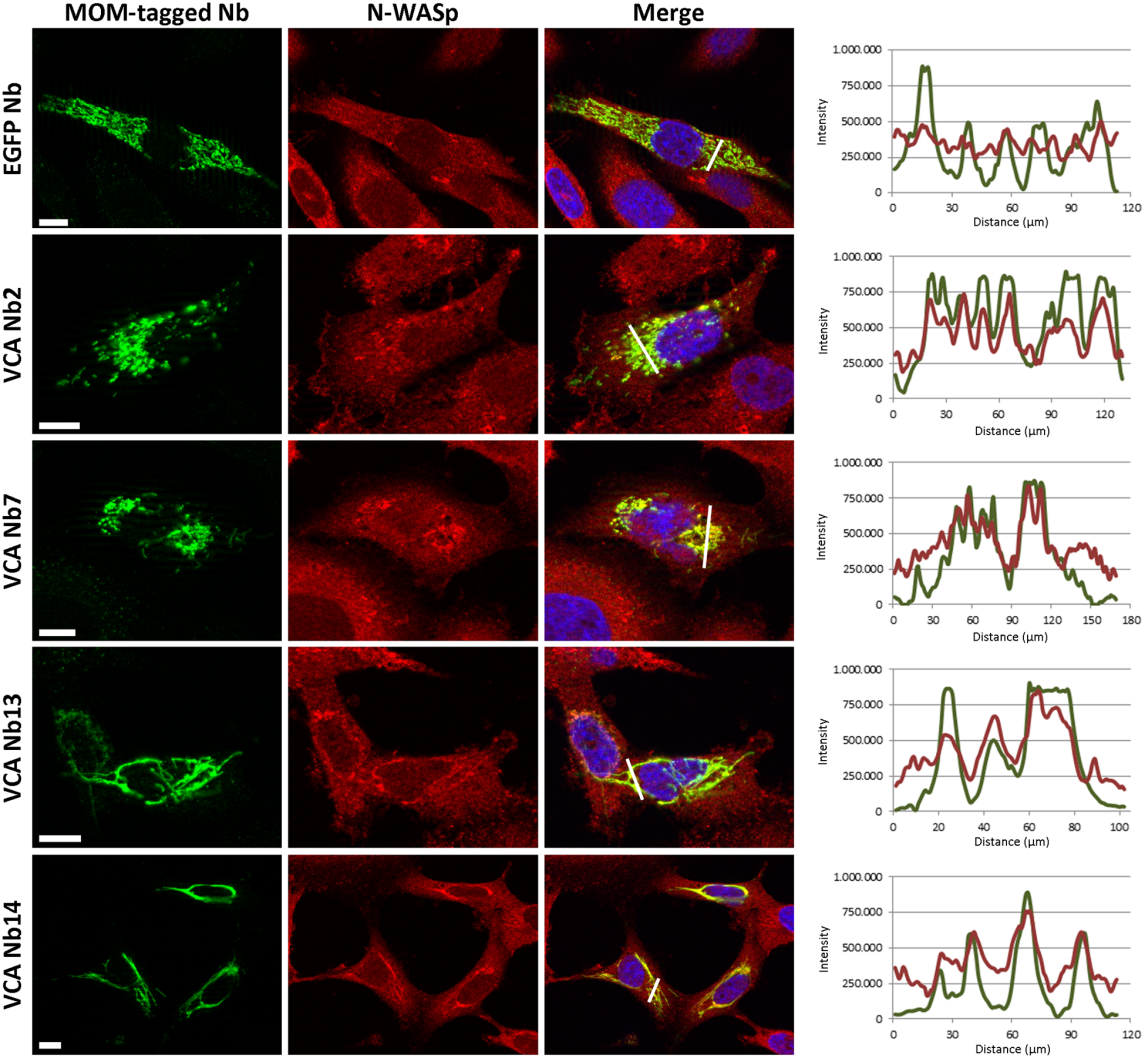
VCA Nb2, 7, 13 and 14 capture endogenous N-WASp at mitochondria. Representative epifluorescence images of MDA-MB-231 breast cancer cells transiently expressing VCA Nbs equipped with a MOM-tag. MOM-tagged EGFP nanobody was used as a negative control (upper panel). Nuclei were visualized with DAPI (blue), nanobodies with anti-V5 antibody (green) and N-WASp with anti-N-WASp antibody (red). Right: the intensity profiles for N-WASp (red) and MOM-tagged nanobodies (green) are very similar, indicative of co-localisation, except for the EGFP nanobody (negative control). (Scale bar = 10 μm).

To investigate whether the nanobodies would still bind their target in the reducing environment of the cytosol, we performed a MOM (mitochondrial outer membrane) delocalisation assay. In this experiment, the nanobodies were equipped with a MOM- and V5-tag at their N-terminus. The MOM-tag originates from yeast TOM70 [34] and redirects the tagged nanobodies to the mitochondrial outer membrane, which can confirm the intracellular binding capacity of VCA Nb to endogenous N-WASp. If N-WASp binds to the VCA Nbs, N-WASp will be found at the mitochondrial outer membrane which does not correspond with its natural subcellular localization. The effectiveness of the MOM-tag is first analysed by performing immunofluorescent assays visualising the mitochondria with Mitotracker Orange (S2 Fig). This confirmed that the MOM-tagged nanobody patterns are similar to the mitochondrial patterns. Further, we will only look at the nanobody patterns as it stands for the mitochondrial patterns. Secondly, the N-WASp co-localisation is performed by visualising the VCA Nbs and N-WASp at the same time. In MOM-tagged EGFP Nb expressing control cells, no mitochondria-like pattern for N-WASp could be found (Fig 2, upper panel). The same was observed in MOM-VCA Nb1, 5 and 15 expressing cells. These are the same 3 nanobodies which did not bind N-WASp in pull down experiments. All other MOM-VCA nanobodies resulted in a mitochondrial-like N-WASp distribution pattern. This was most obvious in MOM-VCA Nb2, 7, 13 and 14 expressing cells (Fig 2, lower panels). The intensity plots were determined as well to quantify the co-localisation (Fig 2, at the right). In conclusion, the nanobodies which were able to bind N-WASp *in vitro* (Fig 1) are also able to efficiently capture endogenous N-WASp in the dense intracellular environment, demonstrating their *in vivo* interaction with the target.

The mitochondrial patterns of the MOM-tagged VCA Nb13 and VCA Nb14 show a dissimilar pattern compared to the control and the other VCA Nb conditions (VCA Nb2 and VCA Nb7) (Fig 2 and S2 Fig). These patterns were only found with the MOM-tag which is used here to demonstrate binding. In all subsequent experiments this tag was not included and under these conditions the mitochondrial pattern looks the same for all nanobodies (S3 Fig).

### Affinity and stoichiometry of 4 selected VCA Nbs

Based on the pull down and MOM delocalisation criteria, we determined the binding affinity and stoichiometric properties of 4 nanobodies; VCA Nb2, 7, 13 and 14. Isothermal titration calorimetry (ITC) was performed at physiological pH by using recombinant VCA Nbs (S4 Fig). As these nanobodies were generated against a domain of N-WASp, we used chemically synthesized pure VCA peptide. The K_d_ values were determined at 0.699 ± 0.154 μM for VCA Nb2, 0.820 ± 0.201 μM for VCA Nb7, 0.685 ± 0.133 μM for VCA Nb13 and 1.597 ± 0.249 μM for VCA Nb14 (Table 1 and S4 Fig). These results showed that VCA Nb2, 7, 13 and 14 have a submicromolar affinity for the VCA peptide. This submicromolar affinity proved to be sufficient as the VCA nanobodies were able to relocate N-WASp to the outer membrane of mitochondria (Fig 2).

**Table 1:** Thermodynamic parameters (and respectively standard deviations) of HA-tagged VCA nanobodies interacting with synthetic VCA peptide, determined by ITC.

Parameters	VCA Nb2	VCA Nb7	VCA Nb13	VCA Nb14
**N**^#^	0.912 ± 0.037	0.905 ± 0.037	0.757 ± 0.035	0.780 ± 0.031
**ΔH (cal/mol)**	-4657 ± 254	-4686 ± 258	-6967 ± 433	-3194 ± 165
**ΔS (cal/mol)**	12.8	12.4	5.2	16.0
ΔG (cal/mol)*	-8537.45	-8445.18	-8551.53	-8044.56
**K_d_ (μM)**	0.699 ± 0.154	0.820 ± 0.201	0.685 ± 0.133	1.597 ± 0.249

^#^Molar ratio or stoichiometry

*ΔG = ΔH−T * ΔS with T = 303.15 K

### The VCA Nbs interfere in the binding between N-WASp and its direct interaction partner Arp2/3, but not actin

**Fig 3:**
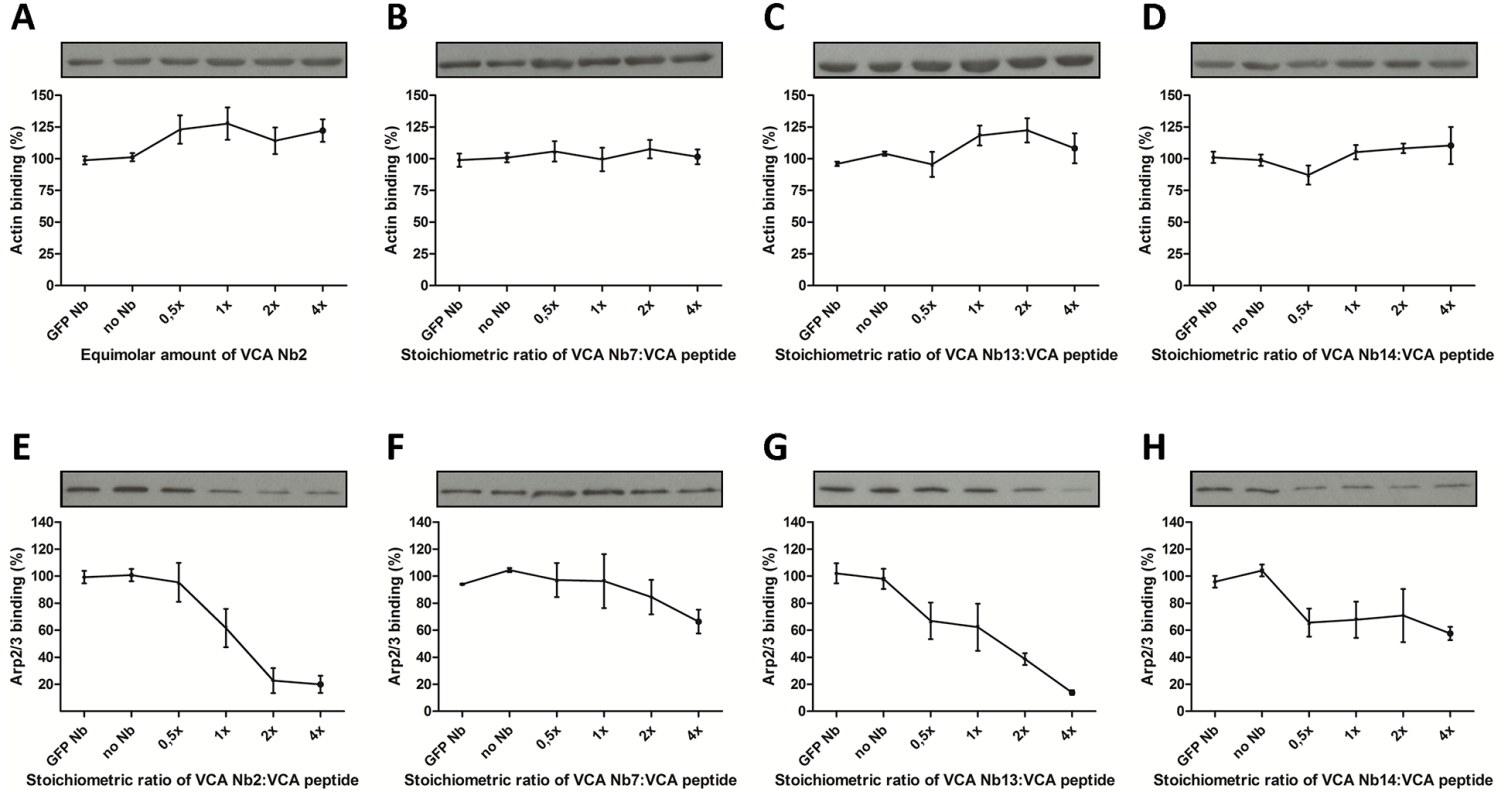
VCA Nb effect on actin or Arp2/3 binding to N-WASp using a nanobody concentration range. Pull down was performed by using biotin-tagged VCA peptide, MDA-MB-231 breast cancer cell lysate, recombinant VCA Nbs and STREPTactin beads. The two controls (no nanobody (No Nb) or EGFP Nb) show maximal Arp2/3 binding. A concentration range was used (VCA Nb: VCA domain stoichiometry of 0.5x, 1x, 2x, 4x). For each repeat, actin as well as Arp2/3 were analysed on western blot and quantification was done using ImageJ (bars represent mean and SEM, n = 3). Kruskal-Wallis and Duns post test was performed. On top the influence of each VCA Nb on actin—N-WASp binding **(A-D)** is shown and at the bottom the effect of each VCA Nb on Arp2/3 –N-WASp binding **(E-H)** is shown.

N-WASps VCA domain can directly interact with actin monomers via its V region and with Arp2/3 via its CA regions. To analyse if the VCA Nbs are able to interfere with the binding between N-WASp and either actin or Arp2/3, a pull down assay was performed by adding a concentration range of VCA Nb2, 7, 13 or 14 together with biotinylated VCA peptide and STREPTactin beads. As an Arp2/3 and actin source, we used MDA-MB-231 cell lysate (Fig 3). Via Western blotting, the amount of bound Arp2/3 and actin was quantified. A quadruplicate of this experiment was analysed with a Kruskal-Wallis test and Dunns post tests. Four different concentrations of each VCA Nb were added, based on the stoichiometry of VCA Nb to the VCA domain. The results show a dose dependent decrease of Arp2/3 binding with increasing concentrations of VCA Nb. While only VCA Nb2 and VCA Nb13 were able to show significant differences, the decreasing trend was found in all four VCA Nbs. This reduction in binding was not observed for actin, meaning that VCA Nbs do not disturb the interaction between N-WASp and actin and VCA Nbs do not interfere at the V region of the VCA domain.

### VCA nanobodies reduce cancer cell invadopodium formation

**Fig 4:**
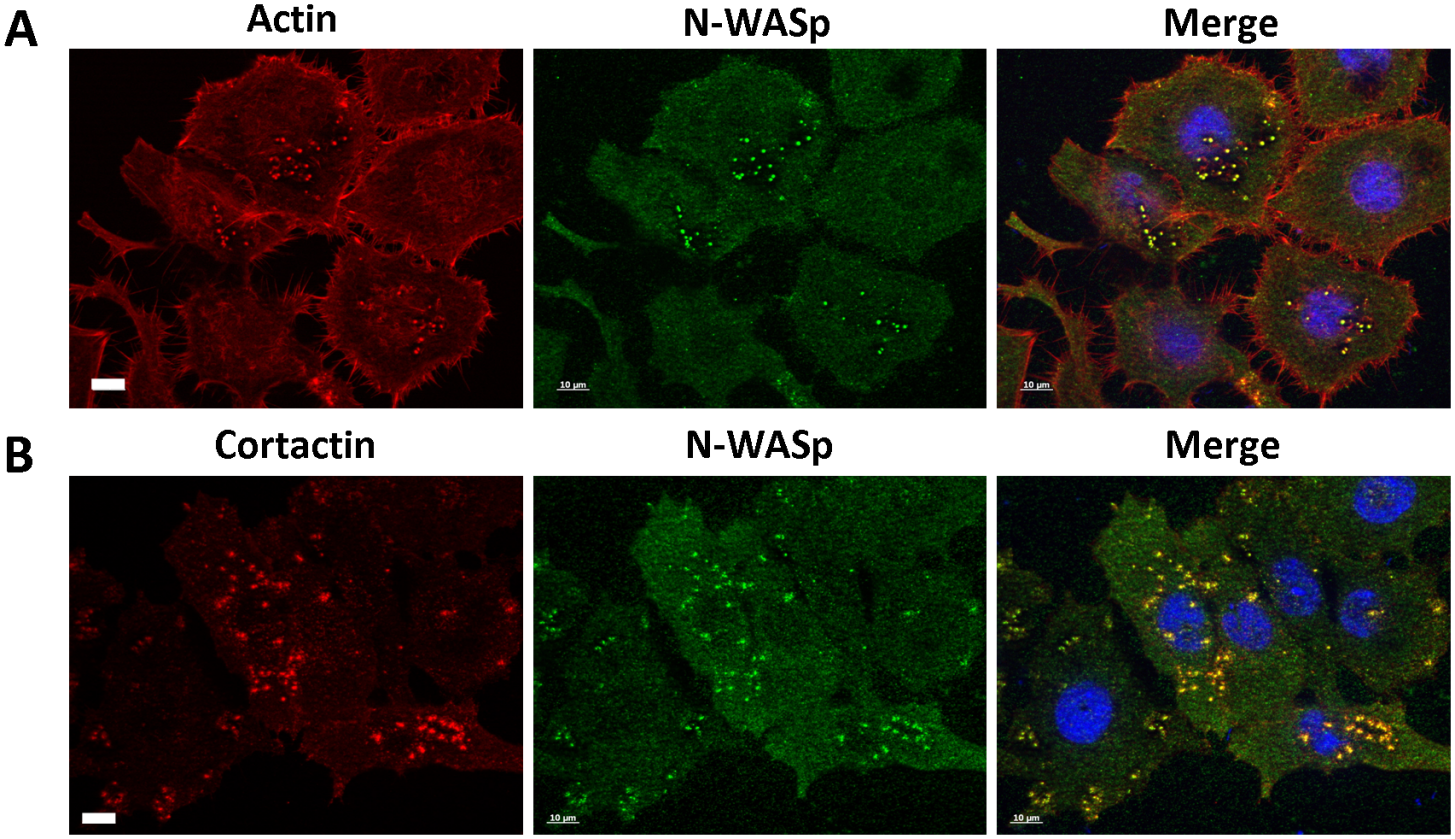
N-WASp colocalizes with invadopodia markers. Representative epifluorescence images of HNSCC61 head and neck squamous cancer cells. Nuclei were visualized with DAPI (blue) and N-WASp (green) with anti-N-WASp antibody. Invadopodia were visualized by using an invadopodium marker; (**A**) actin (red) with Alexa Fluor labelled phalloidin and (**B**) cortactin (red) with anti-cortactin antibody. (Scale bar = 10 μm).

Before a fully mature invadopodium can be formed, an invadopodium precursor has to be created. N-WASp plays an important role in the latter [8–10]. The VCA domain of N-WASp brings Arp2/3 and actin monomers in close proximity to enhance actin polymerisation. This polymerisation creates actin-based protrusions, which in cancer cells may develop into invadopodia. To visualise N-WASp in invadopodia, a staining was performed of invadopodia markers (actin and cortactin) (Fig 4). Our findings clearly revealed N-WASp concentrated at invadopodium sites, which confirms previous research [10, 24].

**Fig 5:**
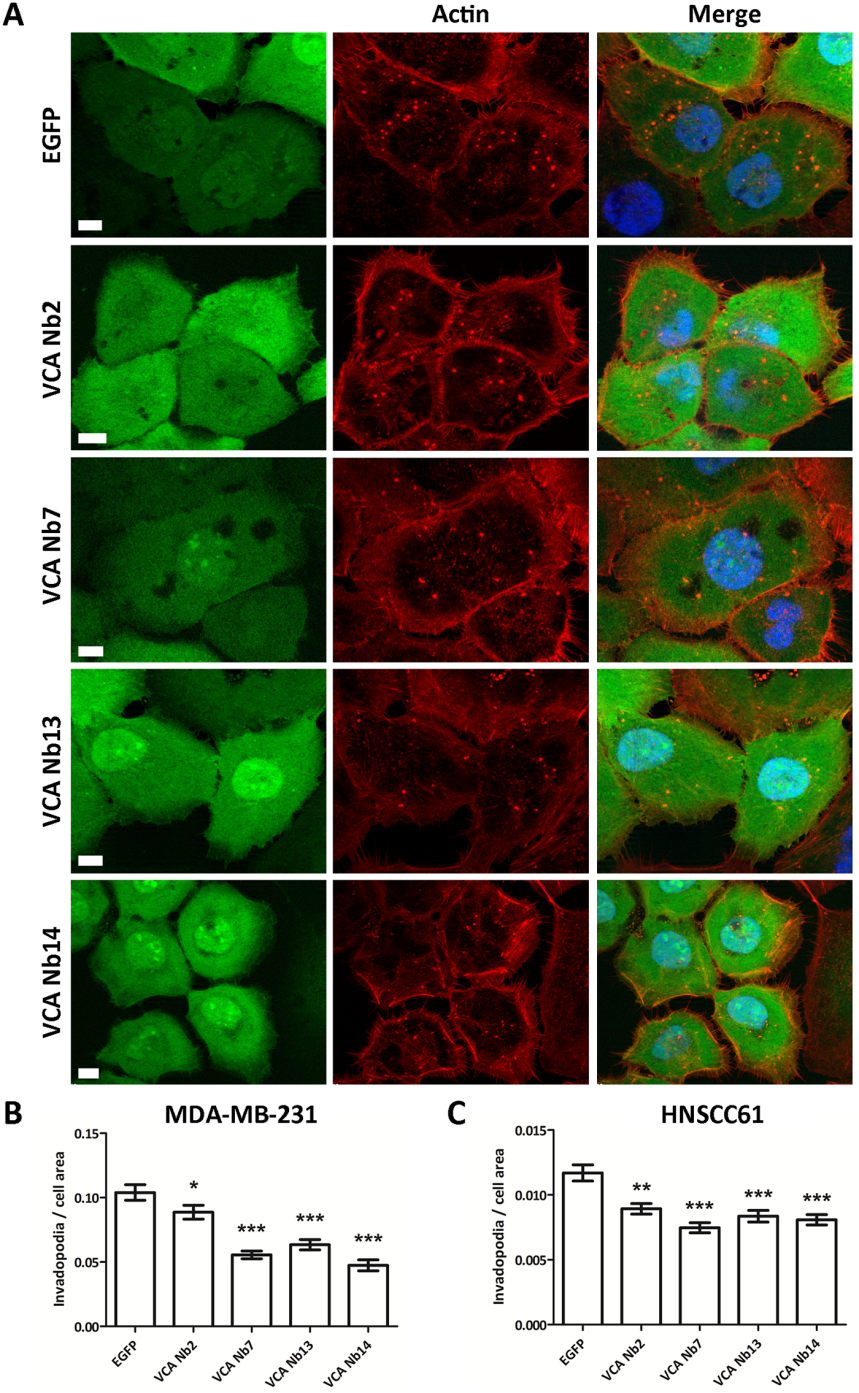
Expression of VCA Nbs in cancer cells reduces invadopodia number. Representative epifluorescence images of HNSCC61 head and neck squamous cancer cells which express EGFP-tagged VCA Nbs (green) (**A**). Nuclei were visualized with DAPI (blue) and actin (red) with phalloidin. Bar plots of MDA-MB-231 breast (**B**) and HNSCC61 head and neck squamous (**C**) cancer cells, which inducible express EGFP- tagged VCA Nbs, were used to count invadopodia per cell. Quantification was done using ImageJ and Kruskal-Wallis and Dunns post tests were performed. As negative control EGFP-only expressing stable cells were used. (* p < 5%, ** p < 1%, *** p < 0.01%).

To analyse if the VCA Nbs could influence invadopodium formation in different cancer cells, lentiviral, stable doxycycline-inducible MDA-MB-231 breast and HNSCC61 head and neck squamous cancer cell lines were made in which EGFP-tagged VCA Nbs are expressed as intrabodies. As a control, an EGFP-only expressing stable cell line was made. After nanobody induction overnight, the cells were fixed with paraformaldehyde and stained with phalloidin. When VCA Nb2, 7, 13 and 14 were expressed a significant reduction in invadopodia numbers was observed in both MDA-MB-231 and HNSCC61 cancer cells (Fig 5).

### The VCA Nbs reduce overall matrix degradation

**Fig 6:**
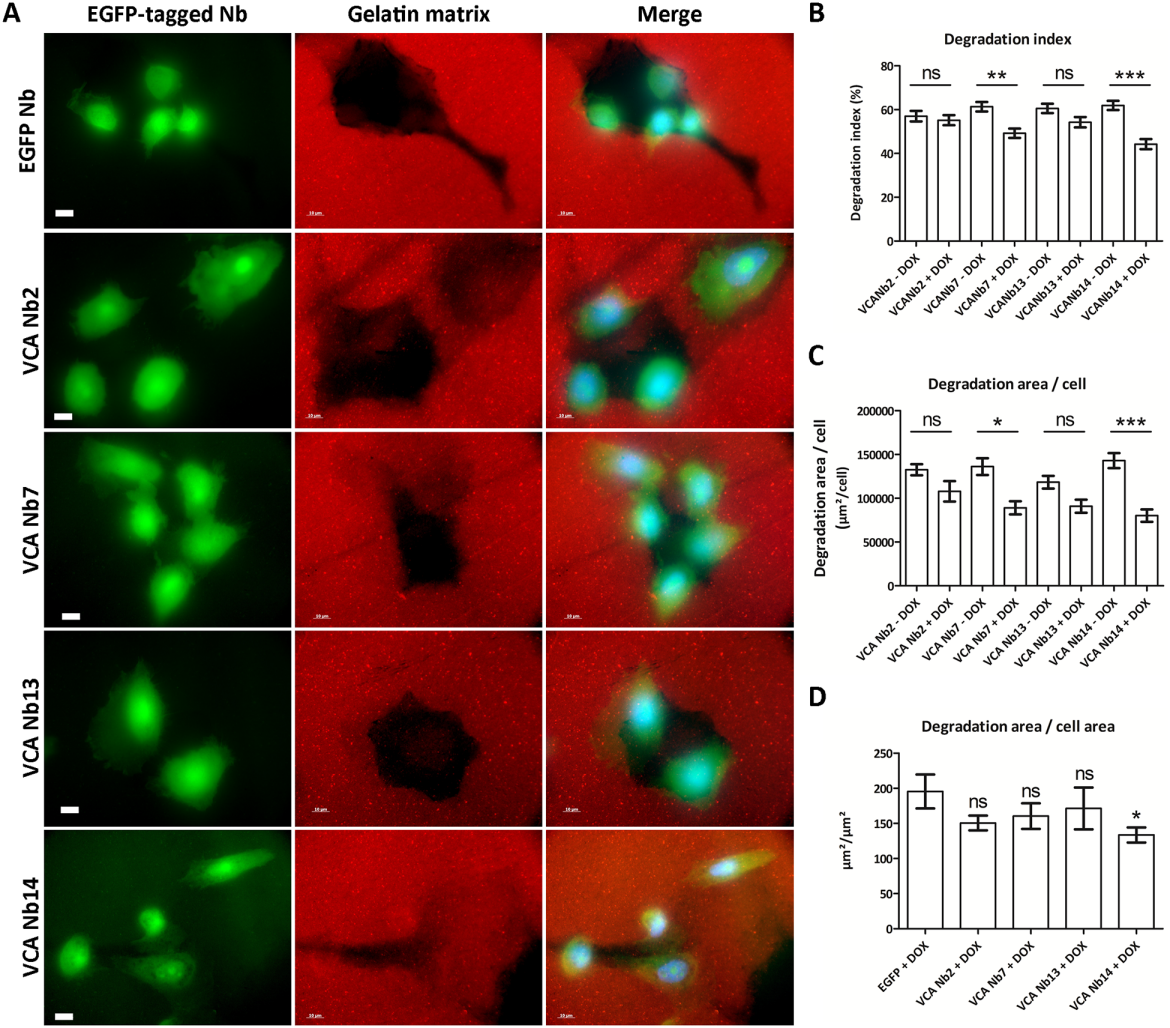
Effects of VCA intrabodies on matrix degradation. (**A**) Representative epifluorescence images of PC-3 prostate cancer cells, which inducibly express EGFP-tagged VCA Nbs, were seeded on Cy3-gelatin matrix (scale bar = 10 μm). As negative control EGFP-only expressing stable cells were used. Quantification was done using ImageJ and Kruskal-Wallis test and Dunns post tests were performed. Degrading capacity of PC-3 cells was analyzed by 3 parameters. For the first parameter ‘degradation index’ (**B**) and the second parameter ‘degradation area per cell’ (**C**), a comparison between ‘not induced’ and ‘doxycycline induced’ was made for each stable cell line. The third parameter ‘degradation area per total cell area’ (**D**) was compared to the EGFP-only cell line. (ns = not significant, * p < 5%, ** p < 1%, *** p < 0.01%).

A conspicuous and important link to metastasis is the proteolytic activity of a mature invadopodium [3]. Once this structure is formed, matrix remodelling follows, underlining the importance of matrix metalloproteinases (MMPs). MMPs are zinc-containing endopeptidases [2, 8, 35, 36]. The gelatinases MMP2 and MMP9 and the membrane bound collagenase MT1-MMP (also known as MMP14) are the most abundant MMPs. The latter is important since it acts as a master switch to activate other MMPs [2, 12, 35, 36]. To analyse the effect of N-WASp nanobodies on matrix degradation, stable doxycycline-inducible PC-3 prostate cancer cell lines were constructed in which the EGFP-tagged VCA Nbs are expressed as an intrabody after adding doxycycline. As a control, an EGFP-only expressing stable cell line was made. In earlier studies we have used a similar approach [27, 28, 37–40]. The cells were seeded on a Cy3-gelatin matrix and overnight induced with doxycycline (Fig 6A). Analysis was done with ImageJ [28, 41] and a Kruskal-Wallis test and Dunns post tests were performed. First, the degradation index was determined. This parameter can be interpreted as the normalised difference between the mean grey value of the background (here the red fluorescent labelled gelatin matrix) and of the cell area. For two out of four VCA Nbs (VCA Nb7 and Nb14), significant less degradation was found (Fig 6B). The second parameter ‘degradation area per cell’ revealed significantly less degradation per cell for the same two VCA Nbs (Fig 6C). Thirdly, the ratio of ‘degradation area to total cell area’ was determined. This parameter gives only for VCA Nb14 a significant reduction in degradation per cell area (Fig 6D). In conclusion, only expression of VCA Nb7 and 14 in PC-3 cells resulted in reduced matrix degradation.

Matrix remodelling is caused by proteolytic activity of invadopodia [3, 8]. The MMPs need to be transported through the invadopodia before they can perform their function extracellularly. N-WASp is suggested to form actin comet tails at vesicles, which brings the MMPs to the membrane [42]. These actin tails will give the vesicles a propelling force to help their movement. To study this, the secretion and activity in the medium of the most abundant MMP was analysed by zymography experiments with PC-3 prostate cancer cells. Initially, the purpose was to study either MMP2 and MMP9, but no MMP2 activity was observed after 20 h of incubation, not even in the control experiment. We could observe a small decrease of MMP9 secretion/activity between the induced and the non-induced VCA Nb-EGFP expressing cell lines but these proved to be not statistically significant (S5 Fig). Furthermore, another proposed function of N-WASp involves proper localisation of MT1-MMP [8]. The potential effect of the VCA Nbs on MT1-MMP positioning was analysed in HNSCC61 cells by counting MT1-MMP containing invadopodia when MT1-MMP positive dots overlapped with F-actin dots. However, no difference in MT1-MMP localisation was observed (S6 Fig). In conclusion, VCA Nb7 and VCA Nb14 reduce the degrading properties of PC-3 cells, but without significant effects on MMP9 secretion/activity or on MT1-MMP localisation.

## Discussion

In this study, nanobodies were generated against the VCA domain of N-WASp, an actin and Arp2/3 interaction partner. Out of all binders, four VCA nanobodies (VCA Nb2, 7, 13 and 14) were selected and expressed in 3 different cancer cell lines. Pull down experiments and immunofluorescence revealed their ability to bind endogenous N-WASp. Their submicromolar affinity for the VCA peptide was determined with ITC. Previous studies found actin binding affinities to the VCA domain of WASP family proteins ranging from 50 to 250 nM, also using ITC [43, 44]. The affinity between VCA and Arp2/3 complex is more difficult to determine and expected to be lower [45–49]. Depending on the techniques used, previous research suggested a binding of one Arp2/3 complex to two N-WASp molecules [45–47]. These previously determined affinities cover a broad range, but almost all conclude that the affinity differences between those two binding sites differs by one or two order of magnitude [45–47]. As we observed a delocalization of N-WASp using the MOM-tagged VCA nanobodies (Fig 2), a decreased binding of Arp2/3 to VCA in the presence of the VCA Nbs (Fig 3), a significant disturbance of invadopodium formation (Fig 5) and a decrease in the overall matrix degradation (Fig 6), we conclude that VCA nanobodies indeed perturb several functions of endogenous N-WASp in these cancer cells.

N-WASp participates in the formation of an invadopodium precursor which activates the actin polymerisation process. It was previously demonstrated that N-WASp is a key player in invadopodium genesis [50]. The cancer cell receives a signal (i.e. EGF) which activates a cascade leading to invadopodium formation. In this cascade, the RhoGTPase CDC42 is activated and will change the conformation of N-WASp from the inactive to the active state [14, 51]. Only in the active state will the VCA domain of N-WASp bind Arp2/3 and actin and stimulate actin polymerisation at the pre-invadopodium site. Next, Arp3 binds to cortactin and after the activated N-WASp comes in, the Arp2/3 complex will interact with N-WASp, leading to a higher activation of Arp2/3 and initiation of actin polymerisation [11]. N-WASp interacts with Arp2/3 through the central or cofilin-like (C) and acidic (A) regions of the VCA domain. Of the seven subunits of the Arp2/3 complex, only Arp2 and Arp3 bind to the VCA domain of N-WASp [11, 12]. This double interaction differs in properties and functions [22, 52]. The first interaction occurs at the acidic (A) region of the VCA domain and is important for the affinity between N-WASp and the Arp2/3 complex, but is not essential for Arp2/3 activation. The second interaction involves the central (C) region, which binds Arp2/3 with lower affinity, but it is indispensable for Arp2/3 activation. Our observations show that the VCA nanobodies are able to interfere with this process. The Arp2/3 and actin binding experiments reveal that the VCA Nbs have no effect on actin binding, but the VCA Nbs all show a reduction in Arp2/3 interaction with VCA. Moreover, we observed a significant reduction in the number of invadopodia, both in MDA-MB-231 and HNSCC61 cells, when VCA Nbs were expressed as intrabody (Fig 5), highlighting the importance of the VCA domain of N-WASp in invadopodium formation [6, 17, 22, 23]. Because VCA Nb2 and VCA Nb13 show a significant reduction in Arp2/3 interaction, we suggest that these nanobodies interrupt the interactions via binding to the acidic (A) region of the VCA domain. In contrast, VCA Nb7 and VCA Nb14 elicit only a slight reduction in Arp2/3 interaction, but display a significant effect in the degradation properties of PC-3 cells. We suggest that VCA Nb7 and VCA Nb14 bind the central (C) region of the VCA domain, where they do not disturb the Arp2/3 interaction that much, but more specifically affect Arp2/3 activation. Because VCA Nb2 and VCA Nb13 do not show an effect on overall degradation, we hypothesize that the interference with the acidic (A) part of the VCA domain is enough to significantly reduce the number of invadopodia but not sufficient to show effects in the following degradation step.

After invadopodium maturation, matrix remodelling will follow. Our results revealed that VCA Nb7 and 14 were able to significantly reduce the overall degradation (Fig 6). Presumably, these nanobodies lead to invadopodia that are defective in protrusion through interfering with N-WASp—Arp2/3 interaction. When there are less protrusive invadopodia, the overall degrading properties of cells will decrease because actin polymerisation is what drives invadopodium growth through the matrix, followed by matrix degradation.

The properties of N-WASp in this pathway may also be related to the transportation of vesicles [15, 42, 53]. To elicit matrix degradation, the MMPs must traverse the invadopodium to the extracellular space, where they can exert their function [53]. N-WASp is expected to activate actin polymerisation via Arp2/3 to form actin comet tails at vesicles [42, 53]. This will give a propelling force to enhance the movement of these vesicles through an invadopodium [15, 53]. To study this further, we analysed MMP9 secretion and activity in VCA nanobody expressing cancer cells. However, in PC-3 cells no effects were found (S5 Fig). This is contrary to what we observed for cortactin [40]. Indeed, cortactin nanobodies targeting its C-terminal SH3 or the N-terminal NTA domains significantly reduced MMP9 secretion and activity. FasNb5 however, which blocks the actin bundling activity of the actin bundling protein fascin that is also present in invadopodia, did not [28, 40]. Alternatively, the degrading properties of N-WASp can be attributed to MT1-MMP as suggested previously [8]. Yu *et al*. concluded that N-WASp plays a role in matrix degradation by optimising the organisation and positioning of MT1-MMP, the master switch of other MMPs. At the cytoplasmic tail of MT1-MMP, N-WASp creates an actin network to specifically stabilize MT1-MMP to increase the effectiveness of degradation [8]. Moreover, no difference in MT1-MMP localisation was observed in HNSCC61 cells (S6 Fig). Currently, we suggest that the reduction in overall matrix degradation is due to the reduction in Arp2/3 complex binding to N-WASp leading to defective invadopodia. When the VCA Nbs reduce the number of invadopodia per cell, the overall degradation properties will also be lower per cell. Less invadopodia implies less available MT1-MMP (enriched in invadopodia) for degradation. We therefore propose that the effect of N-WASp in matrix remodelling is caused by its regulation of invadopodium formation.

Recently, Fulcher *et al*. succeeded in eradicating endogenous proteins via the proteasome degradation pathway using nanobodies and monobodies [54]. If this approach proves to be efficient for a broad variety of targets (providing that a nano/monobody can be generated or is available), it would be an important new tool to study protein function, one that parallels the advent of RNAi in mammalian cells nearly two decades ago [55]. In this respect, Shaefer *et al*. very recently demonstrated that CRISPR/Cas9 genome editing can trigger hundreds of unintended mutations and deletions in the genome [56]. It is therefore incumbent to continue developing new methodologies aimed at studying protein function in cells and organisms and we expect that proteasome-induced protein knock out represents a valuable and important new technological approach towards this end.

In conclusion, nanobodies against the VCA domain of N-WASp affect invadopodium formation and overall matrix degradation, most likely via regulation of N-WASp—Arp2/3 complex interaction, indicating that this region of N-WASp critically contributes to these processes.

## Supporting information

S1 Fig VCA nanobody—N-WASp binding in EGFP-tagged VCA Nb expressing HNSCC61. The EGFP-tagged VCA Nbs were pulled down using a GFP Ab and Protein G Sepharose beads. N-WASp is detected by N-WASp antibody and the VCA-Nbs by GFP Ab.
(TIF)

S2 Fig Mitochondrial pattern in MDA-MB-231 breast cancer cells which transiently express a VCA Nb equipped with a MOM-tag. Representative epifluorescence images showing the mitochondrial patterns indicating that the MOM-tag directs the nanobody to the mitochondrial outer membrane (compare with the Mitotracker channel). MOM-tagged EGFP nanobody was used as a negative control (upper panel). Nuclei were visualized with DAPI (blue), nanobodies with anti-V5 antibody (green) and the mitochondria with Mitotracker Orange (red). (Scale bar = 10 μm).
(TIF)

S3 Fig Mitochondrial pattern in MDA-MB-231 breast cancer cells in which EGFP-tagged VCA Nbs inducably are expressed. Representative epifluorescence images showing the mitochondrial patterns. Nuclei were visualized with DAPI (blue) and the mitochondria with Mitotracker Orange (red). (Scale bar = 10 μm).
(TIF)

S4 Fig Affinity study of VCA Nbs. **ITC profiles of recombinant HA-tagged VCA Nbs with synthetic VCA peptide of human N-WASp.** VCA peptide was titrated once with VCA Nb7 and once with VCA Nb14. VCA Nb2 and VCA Nb13 were titrated with VCA peptide. The upper panel shows the raw data of heat release in function of time, while the lower panel shows the fitted binding curve of total heat release per injection as a function of the molar ratio.
(TIF)

S5 Fig Effects of VCA Nbs on MMP9 secretion and activity levels. (A) Quantification of MMP9 levels in medium was determined using ImageJ after SDS-PAGE and Western blotting. As control the uninduced cell line was used. (B) Activity was obtained after digestion in 0.1% gelatin gel. Quantification was performed using ImageJ and a Kruskal-Wallis and Dunns post tests were performed. The bars represent mean and SEM (n = 3). (ns = not significant).
(TIF)

S6 Fig Effects of VCA Nbs on MT1-MMP positioning. MT1-MMP containing invadopodia were counted when MT1-MMP dots were overlapping with F-actin dots in HNSCC61 cells, in which VCA Nbs expression could be induced. The number of MT1-MMP containing invadopodia was divided by the total amount of invadopodia for each cell and Kruskal-Wallis and Dunns post tests were used. The bar plot represents mean and SEM (n = 3). (ns = not significant).
(TIF)
