# Supplementary figures and images for: VCA nanobodies target N-WASp to reduce invadopodium formation and functioning
Source: PLoS One. 2017 Sep 22;12(9):e0185076. doi: 10.1371/journal.pone.0185076 (PMC5609757; doi:10.1371/journal.pone.0185076)

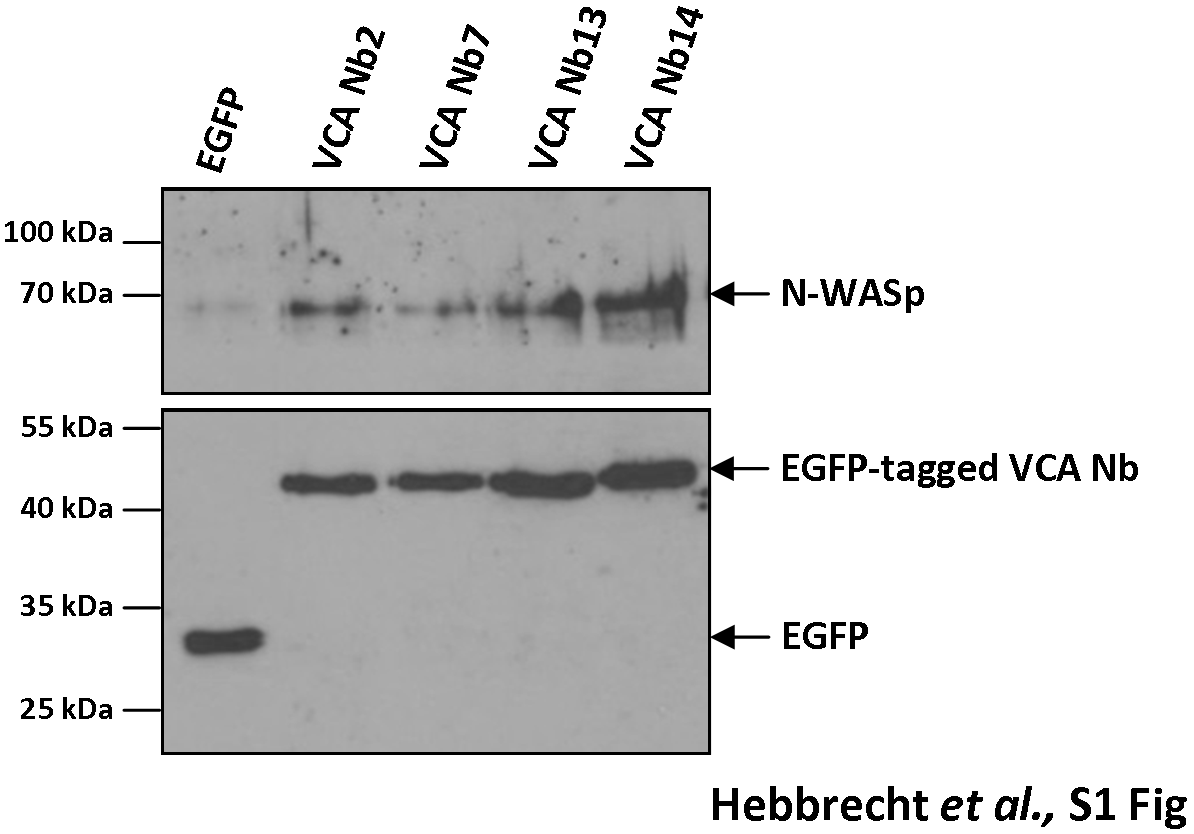

Supplement: S1 Fig — The EGFP-tagged VCA Nbs were pulled down using a GFP Ab and Protein G Sepharose beads. N-WASp is detected by N-WASp antibody and the VCA-Nbs by GFP Ab. (TIF) [file pone.0185076.s001.tif]

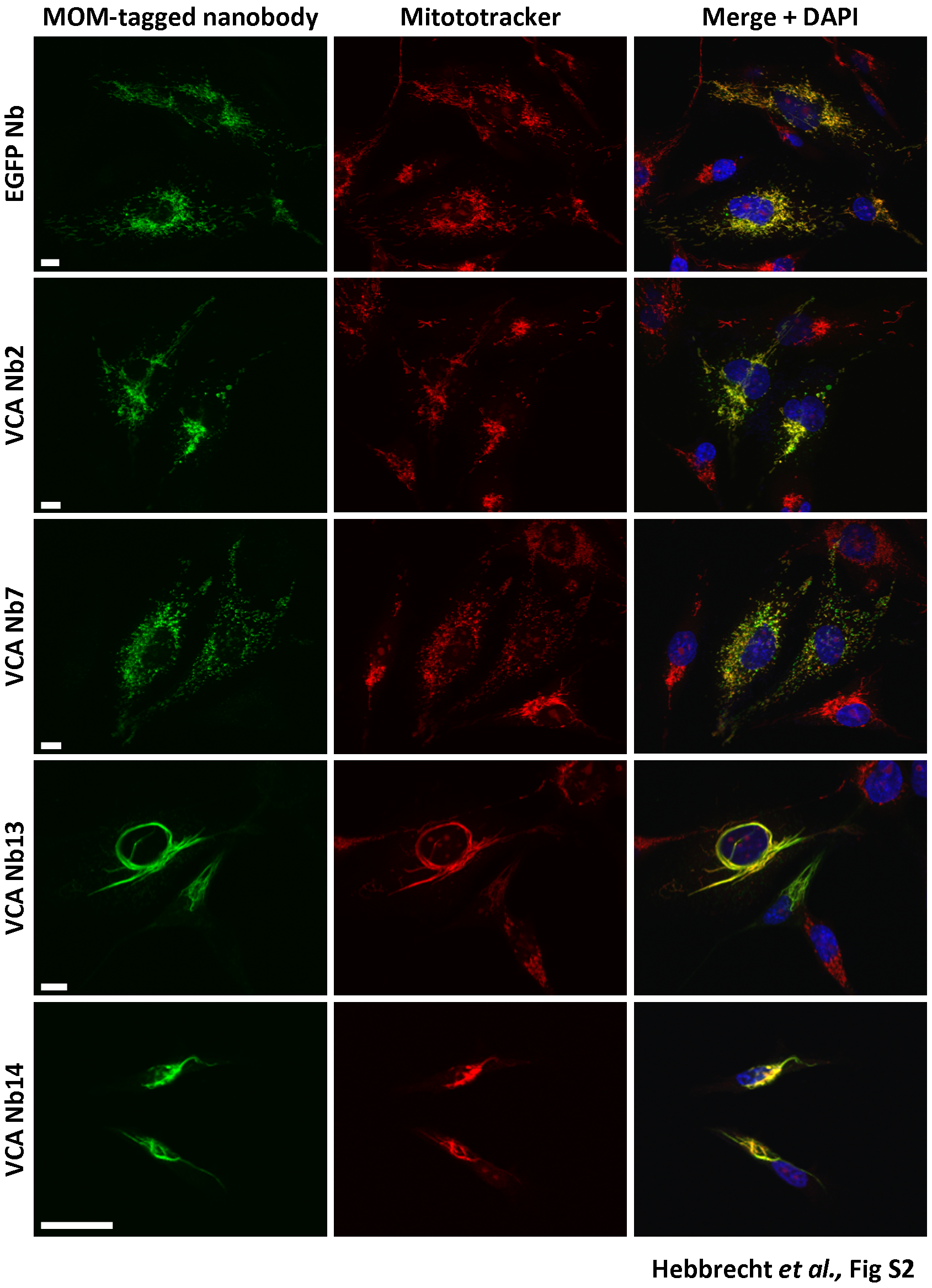

Supplement: S2 Fig — Representative epifluorescence images showing the mitochondrial patterns indicating that the MOM-tag directs the nanobody to the mitochondrial outer membrane (compare with the Mitotracker channel). MOM-tagged EGFP nanobody was used as a negative control (upper panel). Nuclei were visualized with DAPI (blue), nanobodies with anti-V5 antibody (green) and the mitochondria with Mitotracker Orange (red). (Scale bar = 10 μm). (TIF) [file pone.0185076.s002.tif]

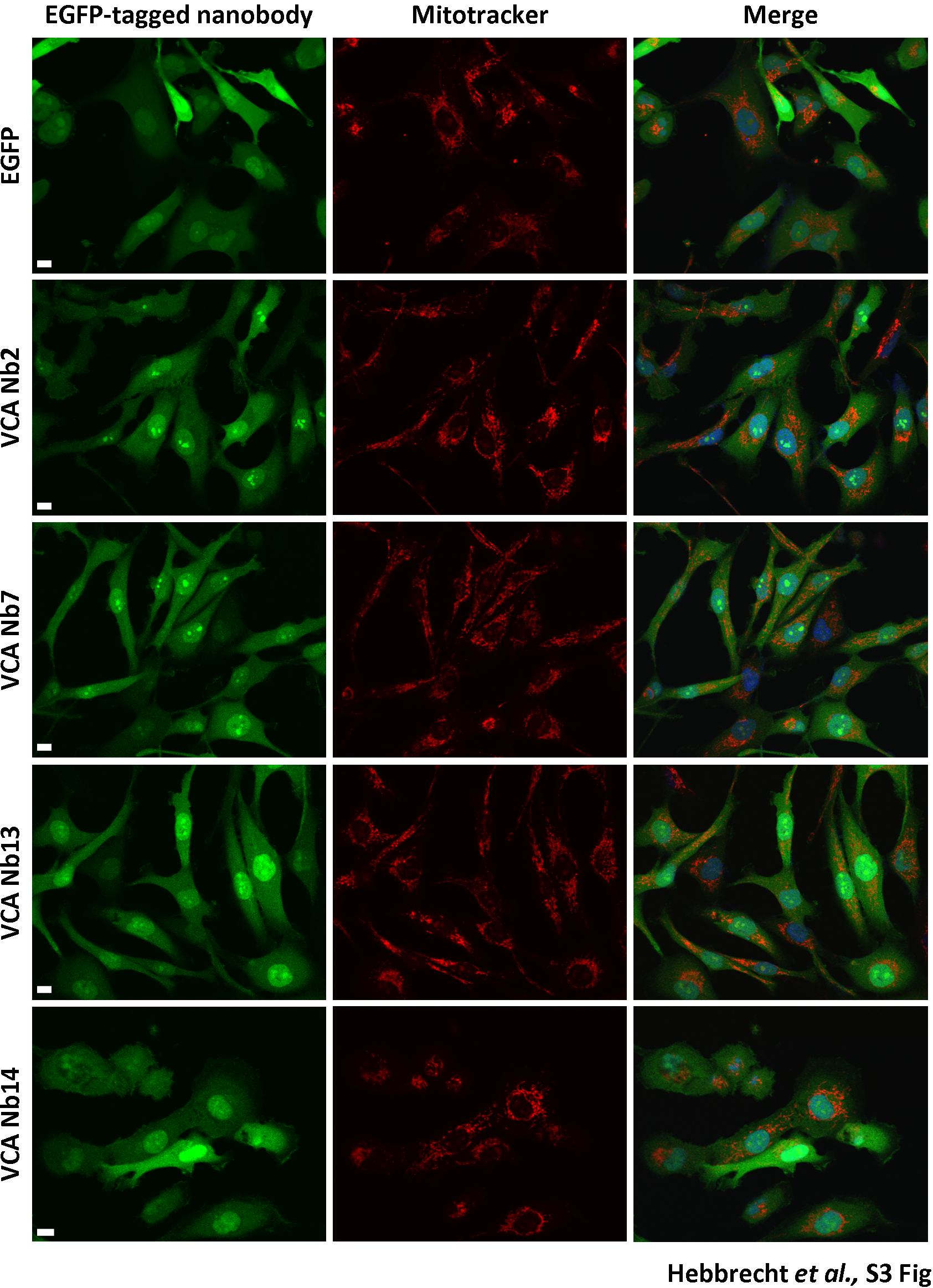

Supplement: S3 Fig — Representative epifluorescence images showing the mitochondrial patterns. Nuclei were visualized with DAPI (blue) and the mitochondria with Mitotracker Orange (red). (Scale bar = 10 μm). (TIF) [file pone.0185076.s003.tif]

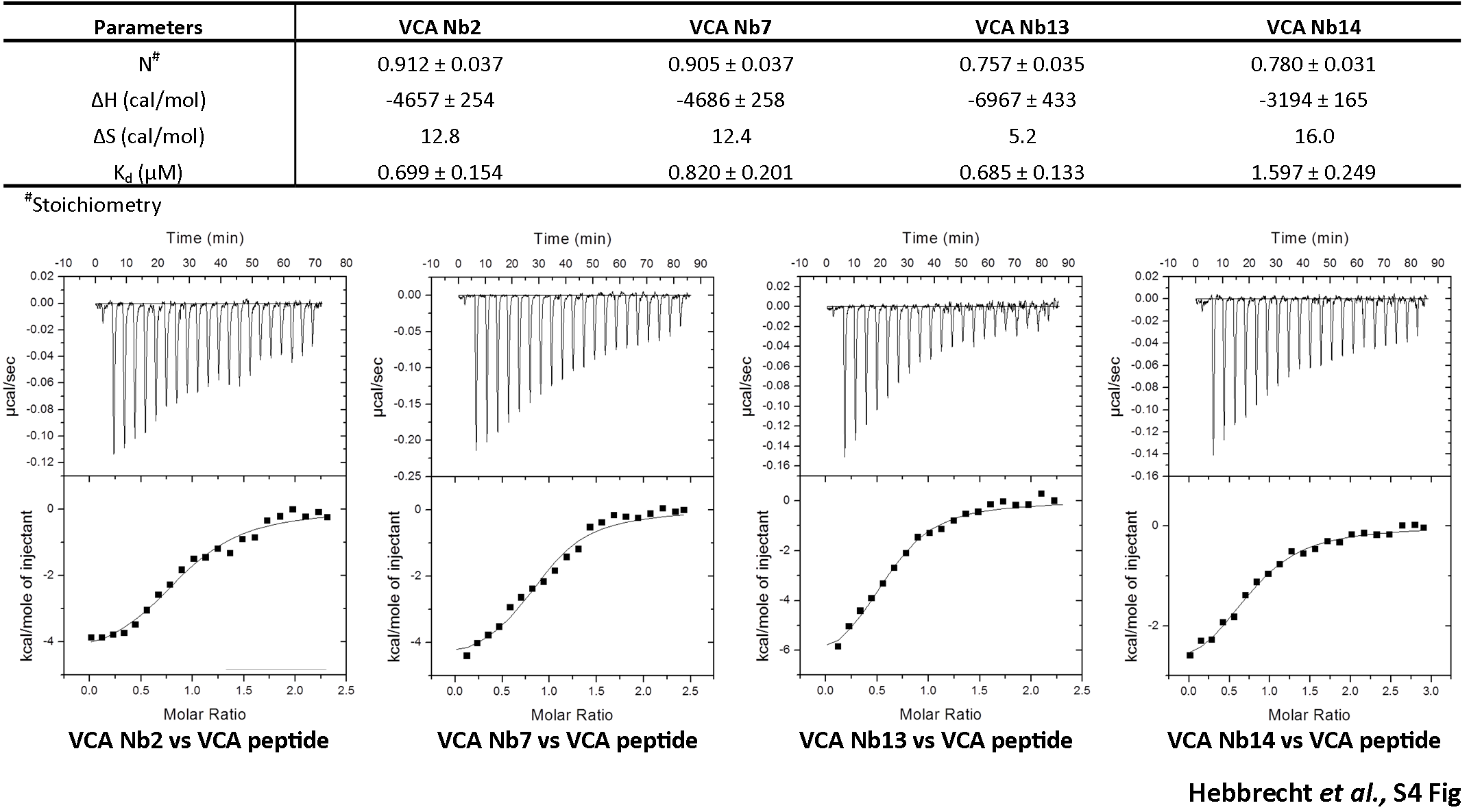

Supplement: S4 Fig — ITC profiles of recombinant HA-tagged VCA Nbs with synthetic VCA peptide of human N-WASp. VCA peptide was titrated once with VCA Nb7 and once with VCA Nb14. VCA Nb2 and VCA Nb13 were titrated with VCA peptide. The upper panel shows the raw data of heat release in function of time, while the lower panel shows the fitted binding curve of total heat release per injection as a function of the molar ratio. (TIF) [file pone.0185076.s004.tif]

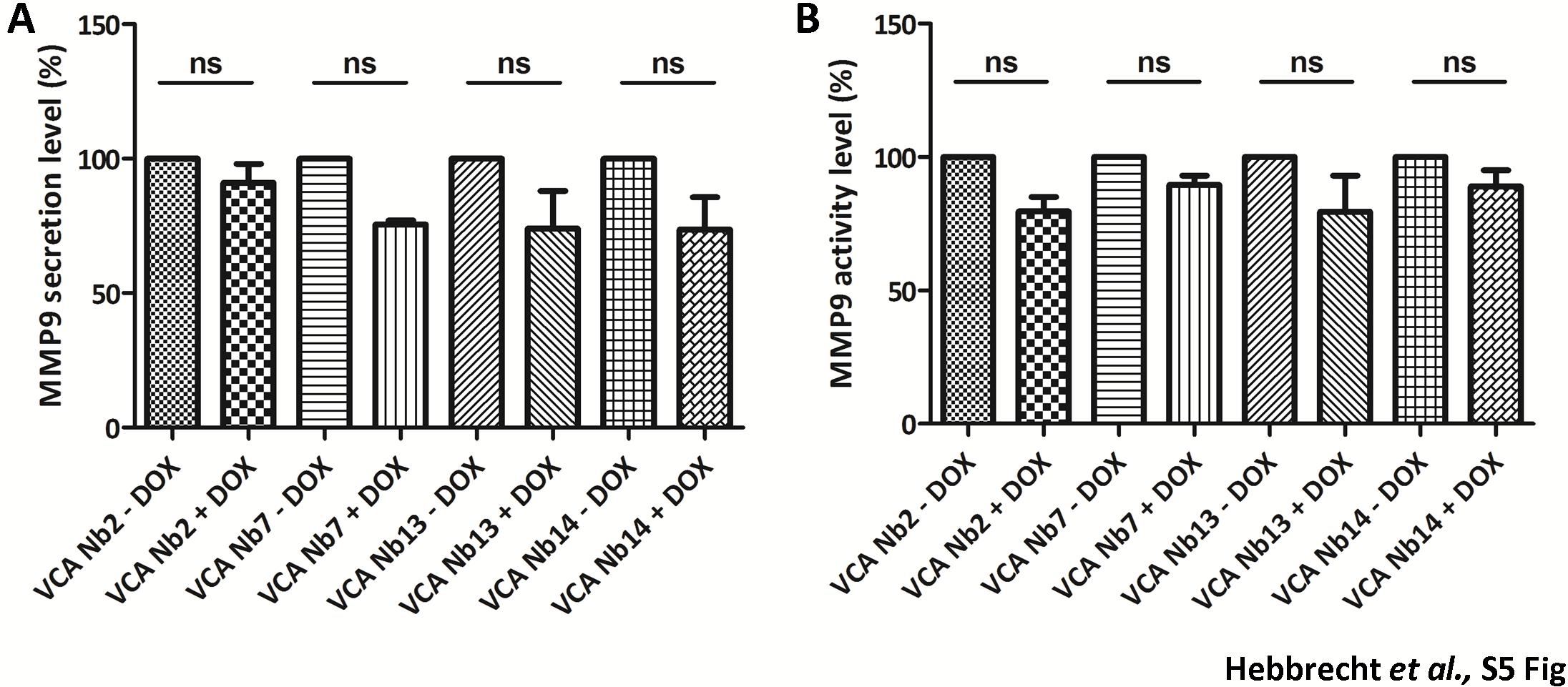

Supplement: S5 Fig — (A) Quantification of MMP9 levels in medium was determined using ImageJ after SDS-PAGE and Western blotting. As control the uninduced cell line was used. (B) Activity was obtained after digestion in 0.1% gelatin gel. Quantification was performed using ImageJ and a Kruskal-Wallis and Dunns post tests were performed. The bars represent mean and SEM (n = 3). (ns = not significant). (TIF) [file pone.0185076.s005.tif]

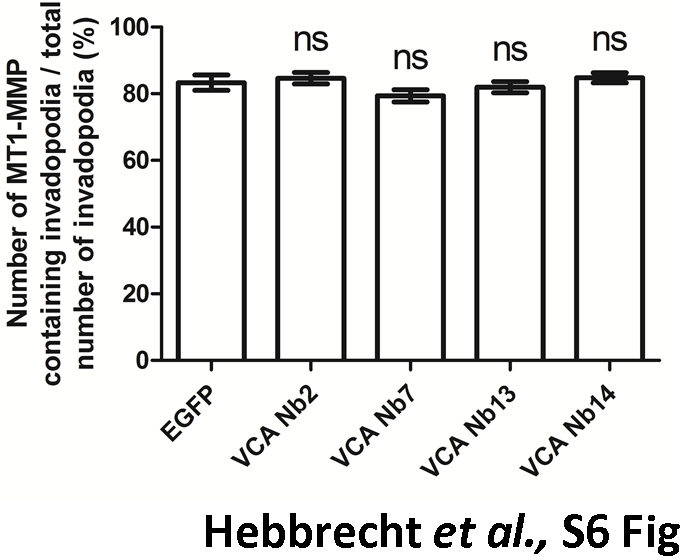

Supplement: S6 Fig — MT1-MMP containing invadopodia were counted when MT1-MMP dots were overlapping with F-actin dots in HNSCC61 cells, in which VCA Nbs expression could be induced. The number of MT1-MMP containing invadopodia was divided by the total amount of invadopodia for each cell and Kruskal-Wallis and Dunns post tests were used. The bar plot represents mean and SEM (n = 3). (ns = not significant). (TIF) [file pone.0185076.s006.tif]
